# The Silencing of *CCND2* by Promoter Aberrant Methylation in Renal Cell Cancer and Analysis of the Correlation between *CCND2* Methylation Status and Clinical Features

**DOI:** 10.1371/journal.pone.0161859

**Published:** 2016-09-01

**Authors:** Lu Wang, Yun Cui, Lian Zhang, Jindong Sheng, Yang Yang, Guanyu Kuang, Yu Fan, Qian Zhang, Jie Jin

**Affiliations:** 1 Department of Urology, Peking University First Hospital and Institute of Urology, Peking University, Beijing, 100034, China; 2 National Research Center for Genitourinary Oncology, Beijing, 100034, China; 3 Urogenital diseases (male) molecular diagnosis and treatment center, Beijing, 100034, China; 4 National Urological Cancer Center, Beijing, 100034, China; Sun Yat-sen University, CHINA

## Abstract

Cyclin D2 (*CCND2*) is a member of the D-type cyclins, which plays a pivotal role in cell cycle regulation, differentiation and malignant transformation. However, its expression status and relative regulation mechanism remains unclear in renal cell cancer (RCC). In our study, the mRNA expression level of *CCND2* is down-regulated in 22/23 paired RCC tissues (*p*<0.05). In addition, its protein expression level is also decreased in 43/43 RCC tumor tissues compared with its corresponding non-malignant tissues (*p*<0.001). We further detected that *CCND2* was down-regulated or silenced in 6/7 RCC cell lines, but expressed in “normal” human proximal tubular (HK-2) cell line. Subsequently, MSP and BGS results showed that the methylation status in *CCND2* promoter region is closely associated with its expression level in RCC cell lines. Treatment with 5-Aza with or without TSA restored *CCND2* expression in several methylated RCC cell lines. Among the 102 RCC tumors, methylation of *CCND2* was detected in 29/102 (28%) cases. Only 2/23 (8.7%) adjacent non-malignant tissues showed methylation. We then analyzed the correlation of clinical features and its promoter methylation. Collectively, our data suggested that loss of *CCND2* expression is closely associated with the promoter aberrant methylation.

## Introduction

In mammals, loss control of cell cycle is a distinctive feature of human carcinogenesis. The regulation of cell cycle mainly associated with the orderly change of phase-specific protein, such as cyclins, cyclin-dependent kinases (CDKs) and CDK inhibitors [1]. Among these cyclins, the D-type cyclins (D1, D2, D3) are mainly involved in regulation of transition from G1 to S phase during the cell cycle [2]. Their critical function is to associate with CDKs and phosphorylates for activity, then leading to the phosphorylation of the retinoblastoma tumor suppressor protein (*RB*), a critical negative regulator of G1/S transition [3]. The phosohorylation of *RB* inactivates it and leads to the activation of transcription factors such as *E2F* suppressed by *RB*, which then increased the activation of transcription of genes involved in DNA synthesis and induced cells to enter S phase [4]. Owing to their pivotal role in cell cycle regulation, the abnormal and untimely expression of these proteins is likely to disturb cell cycle and promote the carcinoma transformation [5,6].

Cyclin D2/*CCND2*, located at chromosome 12p13, is a member of the D-type cyclin family and mainly mediates the extracellular signaling environment with cell cycle progression [7]. *CCND2* was generally considered as a protooncogene in various tumors, overexpression of *CCND2* has been reported in testicular germ cell tumor cell lines [8], colon cancer [9] and gastric cancer correlating with tumor progression and poor prognosis [5,10]. Although well known for its proliferation-promoting function, D-type cyclins were also shown to have the ability of growth-inhibitory function by inducing a senescence-like phenotype [11] and inhibiting cell proliferation [12]. Several studies have found that loss of *CCND2* expression was observed in breast [6,13–15], lung [15], prostate [16], pancreatic [17] and gastric cancer [18]. *CCND2* has also been reported to be the only one of D-type cyclins, being up-regulated in the conditions of growth arrest, ectopic expression of *CCND2* blocked the progression of cell cycle [12], suggesting that *CCND2* might function as a tumor suppressor gene in a cancer-type dependent manner.

Aberrant promoter methylation of tumor suppressor gene is a common feature of renal cell cancer [19–21] and a promising tool for early diagnosis [21]. Precious research has explained that hypermethylation of promoter region within CpG ialands is associated with the transcriptional silence of several genes in multiple kinds of tumors [22]. An increasing number of studies have demonstrated that a large number of TSGs (tumor suppressive genes) have been found to be inactivated by promoter hypermethylation [19–21]. Indeed, reduced expression of *CCND2* has been reported in various cancers and the mechanism underlying *CCND2* silencing in these mentioned cases is the aberrant promoter methylation [6,14–18]. As is reported in prostate cancer, *CCND2* promoter methylation was more frequently observed in high Gleason score tumors [23] and was associated with tumor development in prostate [24].However, the expression of *CCND2* and the regulation mechanism underlying renal cell cancer has not been reported before.

Owing to the controversial role of *CCND2* in different type of tumors, we aimed to assess the expression of *CCND2* and the status of promoter methylation in RCC cell lines, tumors and adjacent non-malignant tissues. Our results showed that *CCND2* was down-regulated in RCC cell lines and primary RCC tissues. Moreover, the methylation status of *CCND2* was more frequently observed in RCC tissues than adjacent non-malignant tissues. Demethylation treatment of RCC cell lines restored *CCND2* expression, which suggested that epigenetic alteration might be the mainly regulated manner of *CCND2* expression.

## Methods and Materials

The experimental methods and usage of clinical samples were approved by the Ethics Committee of the Peking University First Hospital. All these specimens were diagnosed by two urological pathological pathology physicians with patients’ written consent.

### Samples and cell lines

The RCC cell lines 786-O, A498, CAKI-1, CAKI-2, OSRC, 769P, KOTO3 and HK-2 (a “normal” human proximal tubular cell line) were originally obtained (American Type Culture Collection, VA, USA). These cell lines were routinely maintained in RPMI1640 or DMEM medium with 10% fetal bovine serum (FBS) (Invitrogen, Carlsbad, CA), 1% penicillin G, and 1% streptomycin at 37°C in humidified CO_2_ (5%) incubator.

All the RCC samples (fresh tissues and paraffine section tissues) were obtained from the Urology Department, Peking University First Hospital, Beijing, People’s Republic of China. All the RCC tissues and corresponding non-malignant tissues were collected from radical surgical resection without adjuvant therapy. All these specimens were diagnosed by two urological pathology physicians with patients’ written consent. A 2002 AJCC TNM stage and a Fuhrman nuclear grade were used for the classification of the tumor histopathology.

### DNA / RNA extraction and Bisulfite treatment

For RNA extraction, fresh frozen tissues and cell lines were homogenized in TRI Reagent (Molecular Research Center, Cincinnati, OH) and isolated as previously described [25]. Total genomic DNA of RCC tissues and cell lines was extracted according to the manufacturer’s instruction supplied by QIAamp DNA Mini Kit (Qiagen GmbH, Hilden, Germany). Then sodium bisulfite modification of genomic DNA was carried out as described previously [26].

### Reverse transcriptional PCR and Real-time PCR

To evaluate the mRNA expression of *CCND2* in RCC cell lines, reverse transcription PCR (RT-PCR) with GoTaq^®^ Green Master Mix (Promega, Madison, WI, USA) and Real-time PCR with (HT7500 system, Applied Biosystems) were used according to the manufacturer’s protocol. Then we used Real-time PCR to examine *CCND2* mRNA status in 23 paired RCC tumors and adjacent non-malignant tissues. The primers were: *CCND2*-F: CATGGAGCTGCTGTGCCACG; *CCND2*-R: CCGACCTACCTCCAGCATCC. The RT-PCR reaction condition was 35 cycles (94°C for 30 s, 55°C for 30 s, and 72°C for 30 s). Real-time PCR was done for 45 cycles (94°C for 30 s, 55°C for 30 s, and 72°C for 30 s). GAPDH primers were: GAPDH-F: TCCTGTGGCATCCACGAAACT; GAPDH-R: GAAGCATTTGCGGTGGACGAT, which was used as an internal control.

### Demethylation treatment

Six RCC cells were primarily seeded at a density of 1x10^6^ cells/ml in six-well plate, after overnight culture, cells were treated with 10μM demethylation agent 5-Aza (5-aza-2’-deoxycytidine) for 96 hours, some RCC cell lines were further treated with 100 nmol/L histone deacetylase inhibitor TSA (trichostatin A) for additional 24 hours. After the treatment, the cells were collected for DNA and RNA extractions.

### Methylation specific PCR (MSP) and bisulfite genomic sequencing (BGS)

MSP and BGS were performed on the bisulfite-modified DNA samples using primer sets targeting the CpG-rich region in *CCND2* promoter to indentify the *CCND2* promoter methylation status. The methylated and unmethylated primers of *CCND2* were *CCND2*-m1: TACGTGTTAGGGTCGATCG; *CCND2*-m2: CGAAATATCTACGCTAAA-CG (276-bp product); *CCND2*-u1: GTTATGTTATGTTTGTTGTATG; *CCND2*-u2: TAAAATCCACCAACACAATCA (223-bp-product). The MSP was conducted in a 12.5μl reaction solution with GoTaq^®^ Green Master Mix (Promega, Madison, WI, USA) for 35 cycles (94°C for 30 s, 55°C for 30 s, and 72°C). PCR products were analysed in 2% agarose gels. To further confirm our MSP results, BGS was used to analyze the detailed methylation status. The BGS primers were *CCND2*-BGS1: GGAGGAAGGAGGTGAAGAA; *CCND2*-BGS2: CCCCTACATCTACTAACAAAC. 40 cycles/58°C annealing temperature of PCR were performed using GoTaq^®^ Green Master Mix. The PCR products were connected into the pEASY-T5 zero vectors (TransGen Biotech Co., Ltd, Beijing), then 5–9 colonies were randomly selected and sequenced.

### Immunohistochemistry staining

Immunohistochemistry was carried out on forty-three paraffin-embedded RCC tissues and paired adjacent non-malignant tissues microarray with the primary antibody *CCND2* (NOVUS, NBP2-14460) in 1:250 dilution. All the photographs were taken randomly and measured using Image Pro Plus (IPP, version 6.0, Media Cybernatics, Silver Spring, MD, USA).

### Cell transfection

pLVX-IRES-ZsGreen1 and pLVX-IRES-ZsGreen1-*CCND2* vectors were bought from company (YouBio, Chang Sha). Then 786-O and OSRC cells were transfected with pLVX-IRES-ZsGreen1 and pLVX-IRES-ZsGreen1-*CCND2* using Lip 3000 kit (Invitrogen) according to the manufacturer’s instruction.

### Cell proliferation assay

Cells transfected with *CCND2* and vector were harvested and then plated in 96-well plate at a density of 2000 per well and incubated overnight at 37°C. The Cell Counting Kit-8 (Dojindo, Kumamoto, Japan) was used to analyze the cell proliferation. 10μL CCK-8 solution was added into each well with 100μL serum-free optional medium and incubated for 40 minutes. The absorbance was measured at 450nm. All the results were obtained from three independent experiments.

### Statistical analysis

Data were presented as mean ± standard deviation (SD). Student’s *t* test, Fisher’s exact test and Chi-square test were used. Statistical analyzed using the SAS version 9.0 software. A *p*-value was considered significant when less than 0.05.

## Results

### Decreased expression of *CCND2* in RCC patients

**Fig 1:**
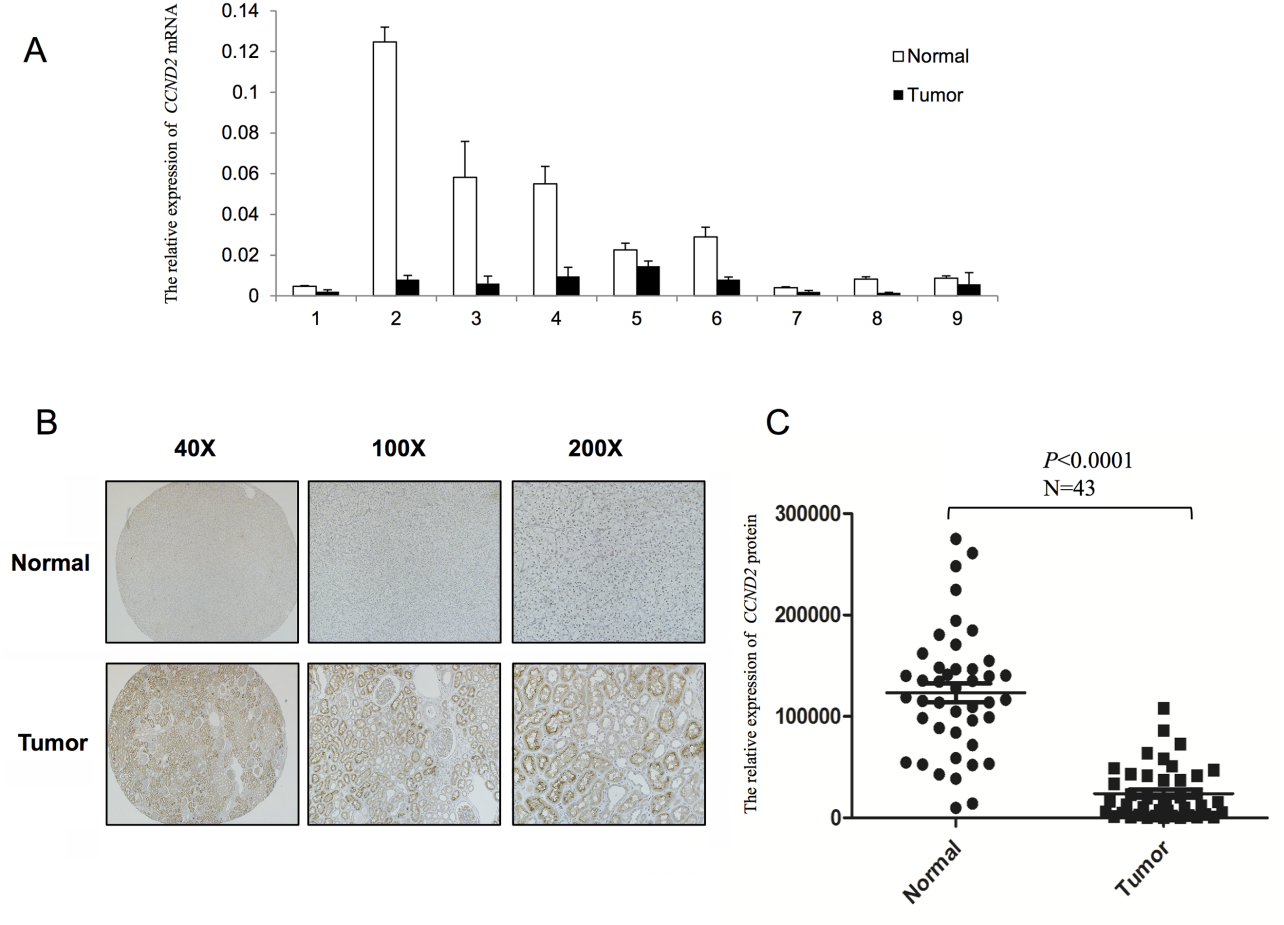
Evaluation of *CCND2* expression in RCC samples. (A) Real-time PCR revealed the mRNA expression level of *CCND2* in paired RCC primary tumor tissues (black column) compared with the adjacent non-malignnat tissues (white column), *p*<0.05; 1–9 represents the patient number (selected randomly from the 23 paired RCC patients). (B) Immunohistochemistry staining showed a significant decrease of *CCND2* protein expression in 43 paired RCC samples in 40X, 100X, 200X magnification. (C) Quantitative analysis of *CCND2* protein expression status with Image Pro Plus software, *p*<0.001.

Serial analysis of gene expression (http://www.proteinatlas.org/) had previously revealed that, *CCND2* expression level was significantly lower in RCC tissues. To check the validity of these findings, we performed Real-time PCR to evaluate the mRNA status of *CCND2* in 23 paired RCC tumors and adjacent non-malignant tissues. The expression of *CCND2* was noted in 22/23 adjacent non-malignant tissues. In contrast, only 1/23 adjacent non-malignant tissue showed slightly decreased expression of *CCND2* compared with RCC tumor tissue (Fig 1A, *p*<0.05). Moreover, we used immunohistochemical staining to examine the *CCND2* protein expression in 43 RCC tumors paired with adjacent non-malignnat tissues. We found that *CCND2* was downregulated in RCC tissues inordinately. Interestingly, *CCND2* protein expression was decreased in 43/43 RCC tumors compared with the corresponding adjacent non-malignant tissues (Fig 1B). By analyzing the immunohistochemistry staining results with Image Pro-Plus and statistical analysis, *CCND2* expression showed a significant difference between tumors and adjacent non-malignant tissues (Fig 1C, *p*<0.0001). Thus, in both mRNA and protein status, specific loss of *CCND2* was observed.

### Hypermethylation of *CCND2* is associated with transcriptional silencing in RCC cell lines

**Fig 2:**
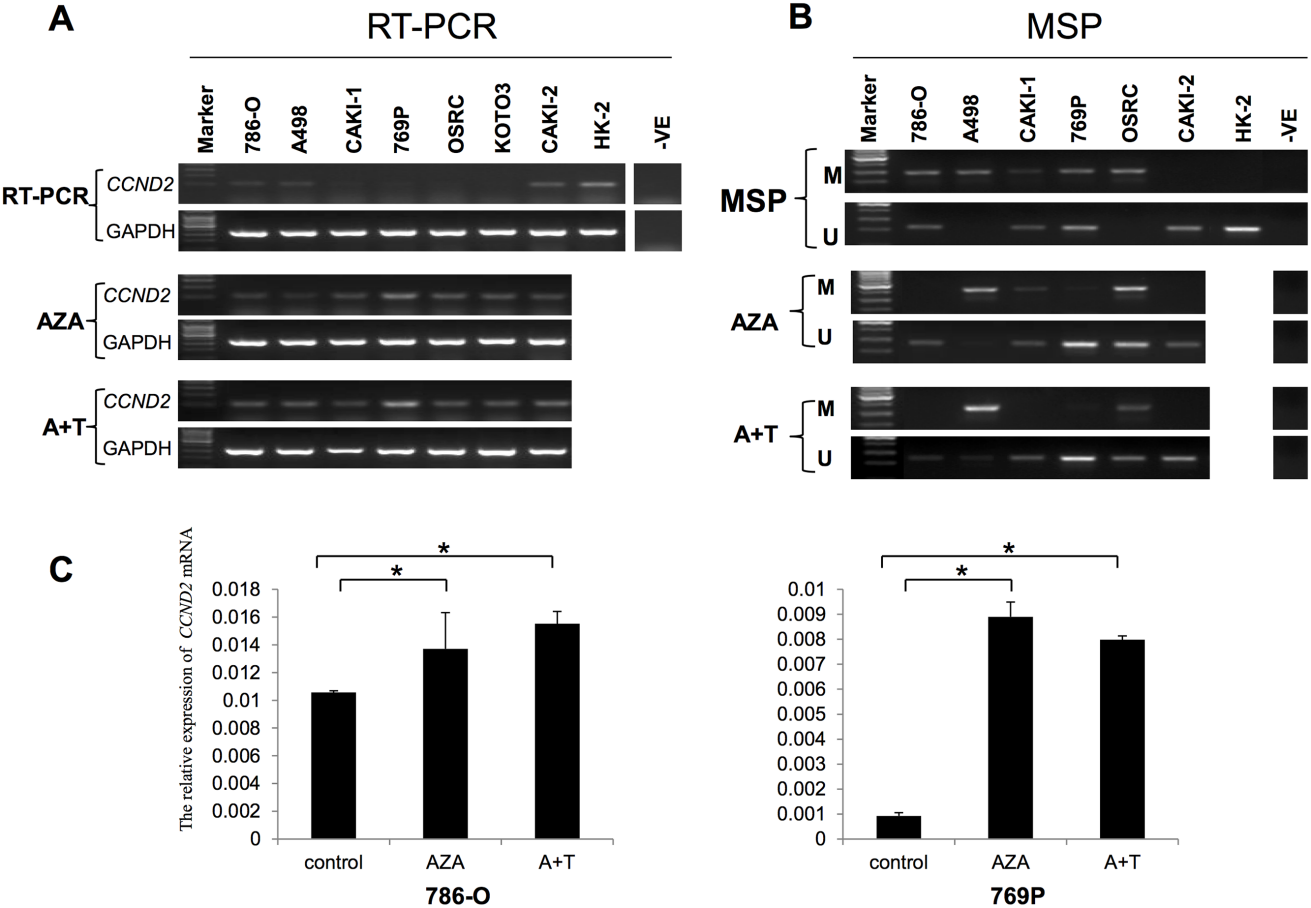
*CCND2* expression and promoter methylation in RCC cell lines before and after demethylation treament. (A) Reverse transcription PCR (RT-PCR) was used to analysis *CCND2* expression in RCC cell lines and HK-2 cell with or without demethylation drugs, AZA: 5-aza-2’-deoxycytidine; A+T: AZA + TSA (trichostatin A); -ve represents negative control. (B) Methylation specific PCR detected the corresponding methylation status in RCC cell lines. (C) Qualitative analysis of *CCND2* mRNA expression in 786-O and 769P cells after demethylation treatment by Real-time PCR, * represents *p*<0.05.

**Fig 3:**
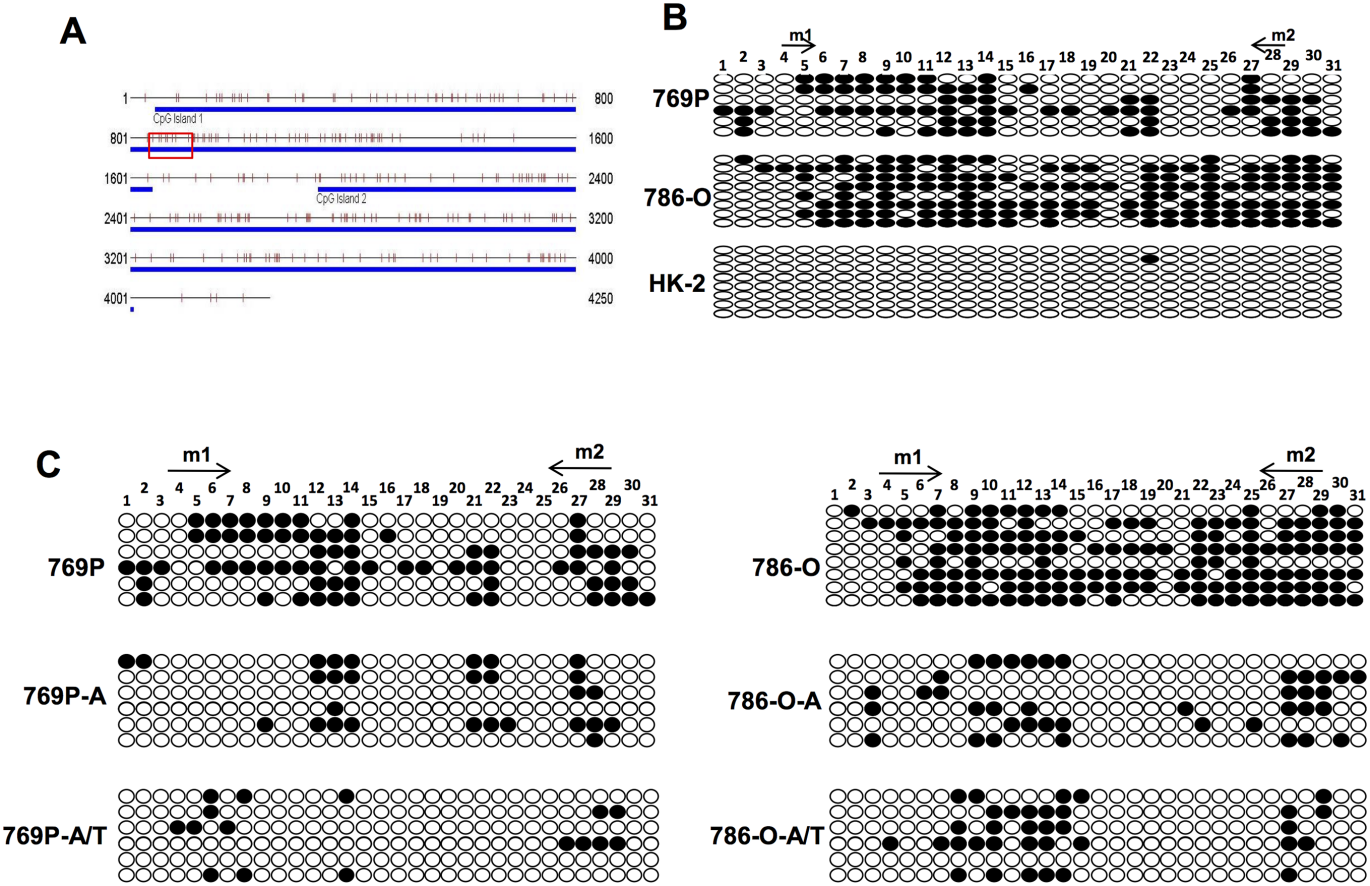
Bisulfite genome sequencing (BGS) analysis of the promoter CpG sites. (A) Two CpG islands were found within *CCND2* promoter region, the red square marked MSP tested sites. (B) Bisulfite genomic sequencing of each CpG site (oval) in *CCND2* promoter region was performed in 769P, 786-O and HK-2 cell lines. Arrows: MSP primers binding sites; Solid oval: methylated CpG site; Hollow oval: unmethylated CpG site. (C) BGS analyzed the methylation of CpG sites after drug treatment in RCC cell lines.

To check the expression of *CCND2* in 7 RCC cell lines and one “normal” human proximal tubular (HK-2) cell line, RT-PCR was used with GAPDH as an internal control. As is shown in Fig 2A, *CCND2* was significantly down-regulated in 6/7 RCC cell lines and weakly decreased in CAKI-2 cell line comparing with HK-2 cell line. This is accordance with the results of RCC clinical samples. As is known to all, both genetic and epigenetic aberrant alteration influence the expression of genes. By analyzing the promoter region, we found two typical CpG islands in *CCND2* promoter region (Fig 3A, http://cpgislands.usc.edu/). To further assess the relationship between epigenetic regulation and *CCND2* expression, methylation specific PCR (MSP) was performed to evaluate the methylation of *CCND2* promoter. Hypermethylation at CpG-rich region with no mRNA expression of *CCND2* was detected in 5/6 RCC cell lines (786-O, A498, CAKI-1, 769P, OSRC), but was not detected in CAKI-2 and HK-2 with *CCND2* mRNA expression (Fig 2B). This implied that promoter methylation might be the mainly mechanism that regulated *CCND2* expression.

Whether the methylation mainly mediated *CCND2* expression needs further exploration. If the down-regulation/silence of *CCND2* was regulated by promoter methylation, then demethylation of the gene by exposure to DNA methyltransferase inhibitor 5-Aza-2'-deoxycytidine (5-Aza) or treatment with histone deacetylase inhibitor trichostatin A (TSA) should result in the up-regulation of *CCND2*. Indeed, when six RCC cell lines were treated with 5-Aza with or without TSA, five out of six cell lines showed a differently increased expression of *CCND2* (as analyzed by RT-PCR and Real-time PCR, Fig 2A and 2C) and partially demethylated (as analyze by MSP, Fig 2B).

### Bisulfite genome sequencing (BGS) analysis of *CCND2* promoter CpG sites

To verify the MSP findings and to explore the extent of promoter methylation, bisulfite genomic sequencing (BGS) was performed. The CpG-rich region of *CCND2* promoter from the nucleotides -1507 to -1130 was sequenced after bisulfite modification. As shown in Fig 3B, BGS was performed in three RCC cell lines: one with *CCND2* expression (HK-2), two with low *CCND2* expression (769-P, 786-O). 769P and 786-O showed higher methylation status than HK-2 cell line, which is consistent with the previously results of MSP. From this part we concluded that hypermethylation may lead to the *CCND2* silencing in RCC cell lines. Meanwhile, BGS results were consistant with the MSP result before or after pharmacological demethylation treatment (Fig 3C). These results implied that aberrant methylation of *CCND2* suppressed its expression in RCC.

### Analyzing the methylation status in primary RCC samples and adjacent non-malignant tissues

**Fig 4:**
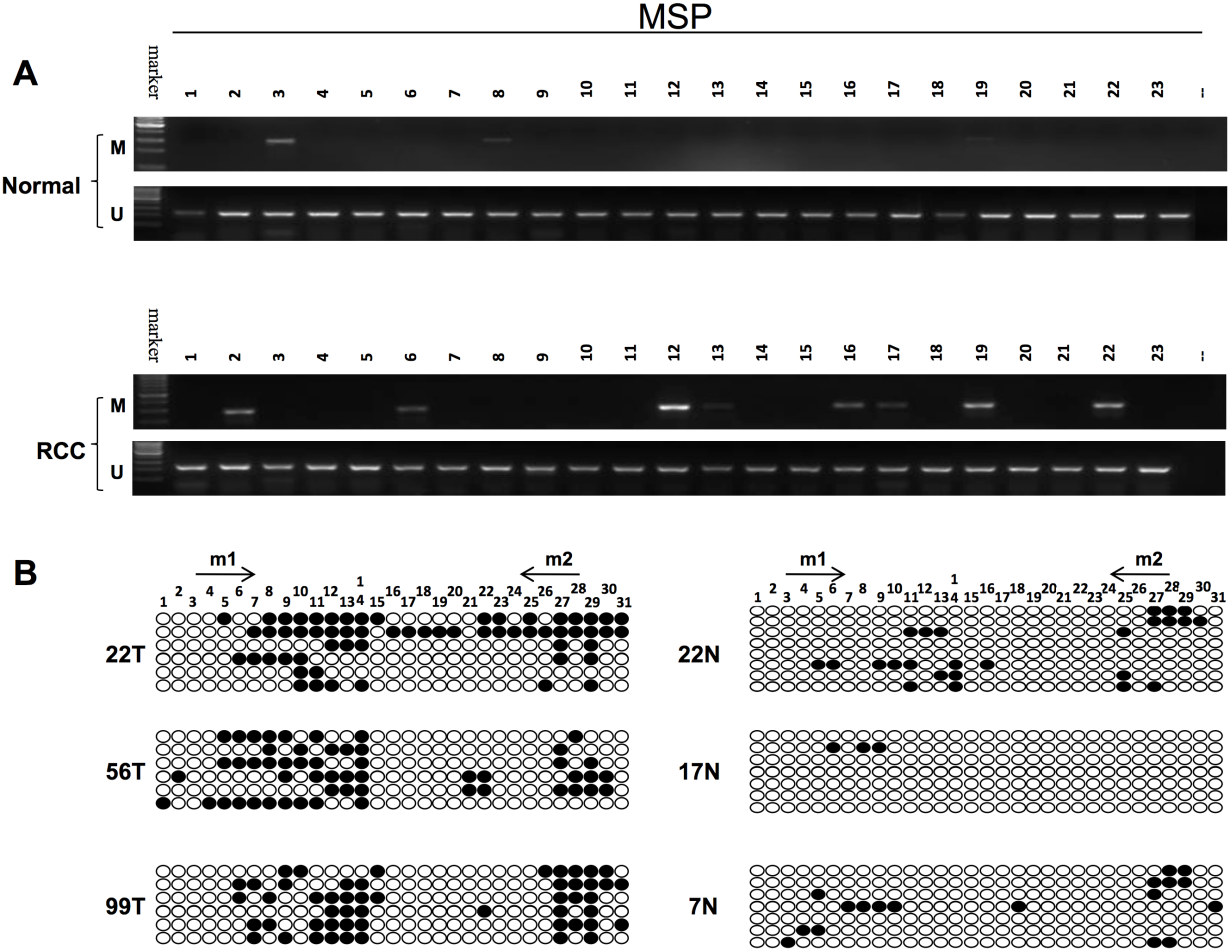
The methylation of *CCND2* in primary RCC samples. (A) MSP analyzed the *CCND2* promoter methylation status in RCC tissues and adjacent non-malignnat tissues, M: metyhlated; U: unmethylated. (B) BGS analysis of *CCND2* promoter CpG sites in RCC patients.

To determine the methylation of *CCND2* promoter, MSP was used to examine the methylation status in 102 RCC tumors and 23 adjacent non-malignant tissues. As is shown in Table 1, the methylation of *CCND2* was observed in 29/102 (28%) of RCC tumors, but only 2/23 (8.7%) in adjacent non-malignant tissues (Table 1), which suggested that *CCND2* methylation was more likely to occur in tumor tissues, as is shown in Fig 4A. To verify the MSP results, BGS was subsequently done (Fig 4B). Then we analyzed the relationship of *CCND2* methylation with clinicopathological features of these patients. As is listed in Table 2, there was no significant difference between *CCND2* methylation and gender, age, tumor site, TNM stage, nuclear grade or histological classification by statistical evaluation. We found the percentage of *CCND2* methylation decreased as the nuclear grade increased, suggesting *CCND2* methylation occurs in a relatively early period in carcinogenesis. In conclusion, *CCND2* methylation is a frequent event in RCC tumorigenesis.

**Table 1:** The percentage of methylation status in RCC samples and adjacent non-malignant tissues.

RCC samples	*CCND2* promoter		Methylation percentage
	Methylation	Unmethylation	
Tumor	29	73	28%
Non-malignant	2	21	8.7%

The methylation of *CCND2* was observed in 29/102 (28%) of RCC, but only 2/23 (8.7%) in adjacent non-malignant tissues.

**Table 2:** The correlation of clinicopathological features and *CCND2* methylation status.

Clinicopathological Features		Methylated NO. (%)	Unmethylated NO. (%)	*p* value
Age		57.34+13.09	55.45+11.9	0.517
Gender	Male	21(33%)	43(67%)	0.2586
	Female	8(21%)	30(79%)	
Side	Left	11(22%)	40(78%)	0.1874
	Right	18(35%)	33(65%)	
TNM classification	pT1	20(30%)	46(70%)	0.0674
	pT2	3(33%)	6(67%)	
	pT3	4(16%)	21(84%)	
	pT4	2(100%)	0(0)	
Nuclear grade	G1	5(38%)	8(62%)	0.6537
	G2	20(27%)	52(73%)	
	G3	4(23%)	13(77%)	
Pathological types	ccRCC	26(30%)	60(70%)	0.49
	Chromophobe RCC	2(33%)	4(67%)	
	Papillary RCC	1(20%)	4(80%)	
	others	0(0)	5(100%)	

Correlation of *CCND2* methylation and gender, age, tumor site, TNM stage, nuclear grade and histological classification, ccRCC: Clear Cell RCC.

### Ectopic expression of *CCND2* inhibits tumor cell proliferation

**Fig 5:**
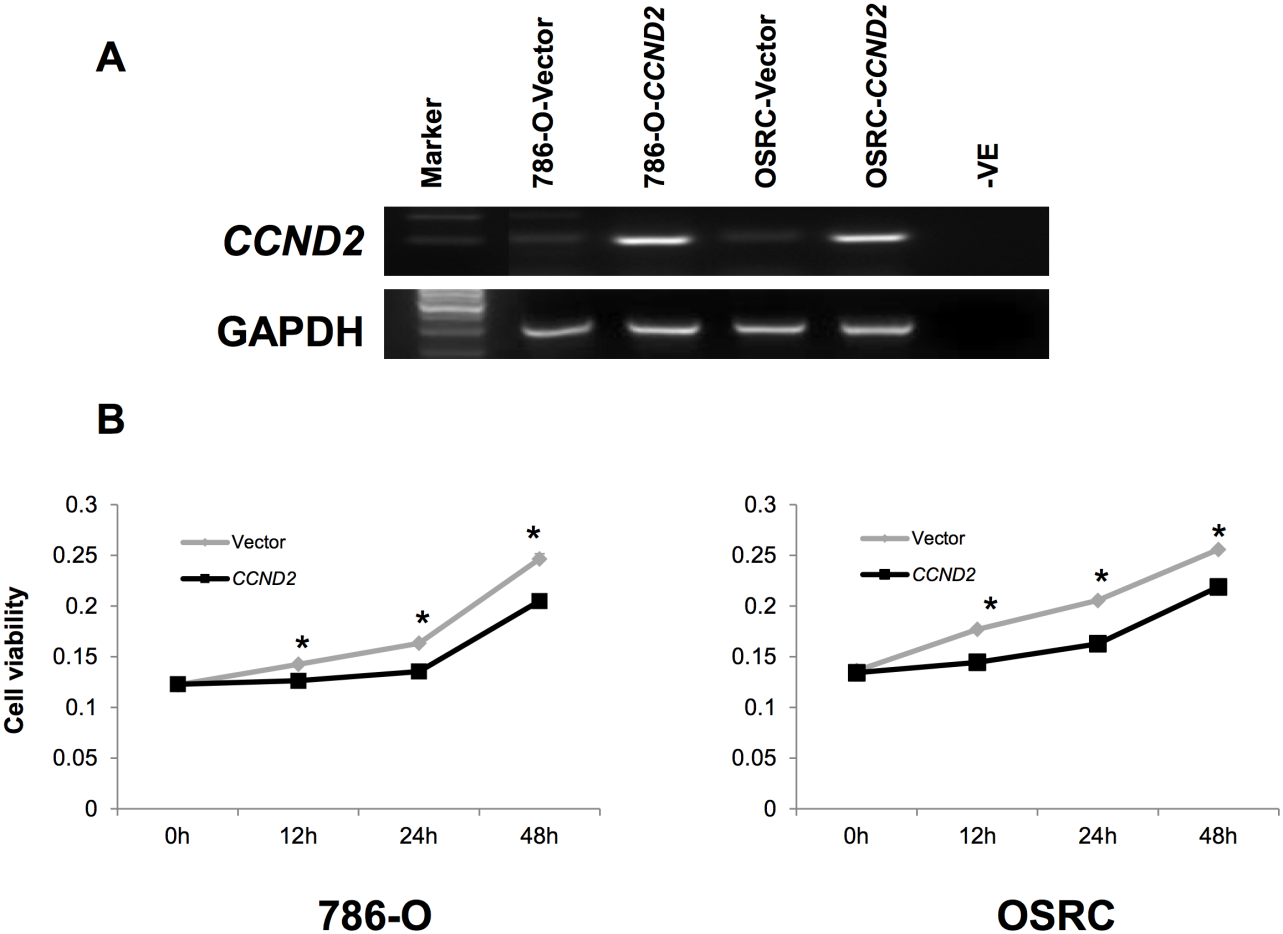
Effect of ectopic *CCND2* expression on tumor growth. (A) Re-expression of *CCND2* in 786-O and OSRC cells was confirmed by RT-PCR. (B) Cell growth curve was inhibited by *CCND2* in 786-O and OSRC cells. The experiments were repeated three times in triplicate. h represents hour Data are mean ± s.d; *p<0.01.

We investigated the effect of ectopic *CCND2* expression on cell proliferation of 786-O and OSRC cells. As the results showed, *CCND2* suppressed the proliferation ability of cells in *CCND2* transfected 786-O and OSRC cells, compared with vector-transfected control cells (Fig 5B). Re-expression of *CCND2* in transient transfected cell lines was evidenced by RT-PCR (Fig 5A). These results implied that *CCND2* could to some extent inhibit the proliferation of tumor cells.

## Discussion

Renal cell cancer is the most common urological cancer which account for about 90% kidney cancer cases in adults with a steady increasing incidence [27]. Even though many advanced methods have been found for RCC diagnosis and therapy, many patients still presented with an aggressive phenotype, including frequent metastasis to distant organs and less sensitive to chemotherapy and radiotherapy [28]. Therefore, elucidation of molecular mechanism underlying RCC and acquisition of specific biomarkers for early diagnosis is urgently needed.

In this study, we found that both RNA and protein expression levels of *CCND2* were decreased in RCC tissues compared to adjacent non-malignant tissues. Moreover, the methylation level of *CCND2* promoter was higher in RCC cell lines than HK-2 cell. It was interesting to found that drug demethylation treatment restored its expression and decreased the methylation level in RCC cell lines. Which suggest that promoter methylation could be the mainly mechanism underlying the loss of *CCND2* expression in both RCC cell lines as well as primary RCC samples.

The cyclin D1-3 protein are generally recognized as regulators that lead to transition of cells from G1 to DNA synthesis by phosphorylation and inactivation of the retinoblastoma protein and activation of cyclin E. Besides for their function in cell cycle, it has been reported that D-type cyclins implicated in the differentiation and carcinogenesis. In renal cell cancer, *CCND1* was absent in normal kidney samples but overexpressed in RCC. High *CCND1* expression was related to good clinical outcome and to most known favorable prognostic factors [29]. Moreover, high *CCND3* protein was observed in 16% of the tumors and was significantly associated with high TNM stage, high nuclear grade, high proliferation and young age [30]. The function and mechanism of *CCND2* has been reported that *CCND2* promote cell cycle progression through phosphorylation of retinoblastoma [3]. On the contrary, *CCND2* could also contribute to the induction and/or maintenance of a non-proliferative state by sequestration of CDK2 catalytic subunit [12]. In spite of this, the role of *CCND2* in renal cell cancer was less thoroughly investigated. Therefore, to assess the *CCND2* expression and the regulation mechanism in RCC is helpful for further understanding the pathogenesis of RCC.

As previously reported, overexpression of *CCND2* has been noted in gastric cancer, as the results showed overexpression of *CCND2* closely correlates with cancer progression and supposed to be an independent prognostic factor [5]. It was also noted that *CCND2* was over-expressed in various kinds of tumors [8,9]. In recent years, several groups have reported the reduced or lack of expression of *CCND2* in breast, lung [6,13–15] and prostate cancers [16]. The different expression of *CCND2* in different type of tumor indicated that *CCND2* may function as an oncogene or TSG in a tumor type-dependent manner. Indeed, overexpression of *CCND2* was linked with increased cell proliferation [31], but it was also reported that *CCND2* could negatively regulate cell growth. Previous studies demonstrated that overexpression of *CCND2* in fibroblast cell efficiently blocked the early G0 and G1 phase [12]. Thus, we showed diminished *CCND2* mRNA expression in 23 RCC samples compared with adjacent non-malignant tissues (*p*<0.05). *CCND2* mRNA was also decreased in 6/7 RCC cell lines as the RT-PCR results showed. Furthermore, lack of protein expression was also observed in 43 paired RCC samples (*p*<0.001). After ectopic expression of *CCND2*, we found the ability of tumor growth was inhibited. These findings may indicate a tumor suppressive role of *CCND2* in RCC, and its down-regulation may be related to tumorigenesis.

Multiple mechanisms underlying inactivation of genes have been reported in various types of cancers, such as loss of heterozygosity, point mutations, homozygous deletions, and aberrant promoter methylation [32]. Aberrant methylation of CpG islands was identified as epigenetic mechanism that regulate the inactivation of TSGs in many cancer types, the percentage of methylated genes in cancers is estimated to be high [33,34]. Multiple TSGs has been reported to be down-regulated by promoter methylation in RCC [19,20]. The transcriptional silencing of *CCND2* by aberrant promoter methylation has been reported in many kinds of tumors. In prostate cancer, the frequency of *CCND2* methylation status was much higher in prostate cancer samples than in non-malignant prostate tissues (*p*<0.005). By analyzing the clinicopathological correlations, greater *CCND2* methylation frequency was found to occur in high Gleason score group and in patients with higher mean PSA value. In addition, methylation of *CCND2* correlates with prostate poor prognosis [23]. Likewise, it was reported that majority of primary breast carcinomas lack expression of *CCND2* mRNA (18 of 24) and protein (10 of 13). In contrast, *CCND2* was expressed in normal luminal and myoepithelial breast tissues. The silencing of *CCND2* expression was associated with its promoter aberrant methylation by MSP. The methylation of *CCND2* promoter was also detected in ductal carcinoma in situ, which suggested that loss of *CCND2* is an early event in tumorigenesis and may associated with the evolution of breast cancer [6]. The absence of *CCND2* expression by promoter methylation is also observed in gastric cancer [18]. To search for a mechanism underlying the consistent loss of *CCND2* in renal cell cancer, we then test the methylation of *CCND2* promoter as a possible cause that lead to the silence of this gene.

As the results showed, the methylation level of *CCND2* was significantly higher in RCC tissues than in adjacent non-malignant samples (Table 2). Moreover, drug demethylation treatment restored *CCND2* expression in RCC cell lines, indicating epigenetic regulation mainly controlled the expression of *CCND2* and *CCND2* promoter methylation may play a role in tumorigenesis. We also analyzed the correlation between *CCND2* methylation and clinicopathological features such as gender, tumor diameters, pathological stage, nuclear grade and histological classification. No association was observed between *CCND2* promoter methylation status and the clinicopathological features (gender, tumor diameters, pathological stage, nuclear grade and histological classification) of RCC patients. Many genes are known to be aberrantly methylated in RCC [19,21,35,36], according to our results, methylated *CCND2* may be an useful part of the panel of markers that could be used for clinical diagnosis. Further exploration is needed to verify our previous results.

## Conclusion

In conclusion, the result that *CCND2* is down-regulated in renal cell cancer strongly suggests that the function of *CCND2* is not only limited to its role in cell cycle transition from G1 to S. The cancer-specific loss of *CCND2* in renal carcinoma and suppression of RCC cell growth provide clues for investigating a possible role of *CCND2* in carcinogenesis in kidney and the methylation of its promoter may function as an optional biomarker for clinical application.
